# Emotion regulation in autistic adolescents: a mixed methods systematic review

**DOI:** 10.1186/s11689-025-09664-y

**Published:** 2025-12-10

**Authors:** Jan Micheel, Holger Zapf, Sarah Hohmann, Carola Bindt, Johannes Boettcher

**Affiliations:** Department of Child and Adolescent Psychiatry, Psychosomatics and Psychotherapy, University Medical Center Hamburg-Eppendorf, Martinistrasse 52, Hamburg, 20246 Germany

**Keywords:** Systematic review, Autism, Emotion regulation, Adolescents

## Abstract

**Background:**

Emotion regulation (ER) difficulties are common in autistic individuals and may contribute to co-occurring psychopathology during adolescence. However, age-group heterogeneity in existing research limits understanding of ER processes in autistic adolescents. Therefore, this mixed methods systematic review synthesizes current knowledge on ER in autistic adolescents aged 10–24 years.

**Methods:**

We systematically searched MEDLINE, PsycINFO, Web of Science, and Scopus for empirical studies on ER in autistic adolescents. 32 studies (including two qualitative) met inclusion criteria and were synthesized using a convergent integrated approach.

**Results:**

Autistic adolescents consistently exhibited more ER difficulties than non-autistic peers, which were associated with internalizing and externalizing symptoms. Greater autism symptom severity, lower theory of mind, and social challenges were frequently linked to lower ER, while no consistent associations with age, gender, or IQ were found. Few studies examined physiological or neurobiological factors, but evidence suggested associations between ER difficulties, lower heart rate variability, and atypical neural responses. Cognitive-behavioral and mindfulness-based interventions generally led to improvements in ER, though results varied and discrepancies between self- and proxy-reports were common.

**Conclusion:**

ER challenges are pronounced in autistic adolescents and are closely associated with mental health symptoms. While interventions show promise, future research should address measurement heterogeneity, examine neurobiological underpinnings, and include more longitudinal and ecologically valid designs.

**Trial registration:**

CRD42024529184 (registered April 06, 2024).

**Supplementary Information:**

The online version contains supplementary material available at 10.1186/s11689-025-09664-y.

As a neurodevelopmental disorder, autism spectrum disorder (ASD) is characterized by challenges in social communication and interaction, as well as restricted and repetitive behaviors [1]. The prevalence of ASD has increased significantly in recent decades [2], a trend partially attributed to heightened awareness of autistic individuals with mild symptoms and without additional cognitive impairments [3]. Moreover, the presence of co-occurring mental health issues, such as anxiety and depression, is more common than exceptional among autistic individuals across the entire spectrum [4] with emotion regulation (ER) discussed as a potential underlying transdiagnostic process contributing to psychopathology [5, 6]. These mental health issues often persist or exacerbate during adolescence [7, 8], complicating the clinical presentation of autism and underscoring the need for tailored interventions that address secondary challenges during this critical developmental period. Given the increasing heterogeneity of ASD diagnoses, it is vital to understand the nuances of ER in autistic adolescents to inform the development of effective support strategies.

ER has been defined as a goal-directed process of monitoring, evaluating, and modifying the intensity, duration, and quality of emotions. This process can involve strategies to enhance positive emotions, diminish negative ones, or maintain emotional balance in response to various situations [9, 10]. ER should be distinguished from related terms such as coping, which emphasizes managing stress responses [11]. The extended process model provides a framework for organizing and analyzing various aspects of ER [11, 12]. It encompasses three stages: (1) Identification, which involves recognizing the need for regulation and becoming aware of emotional experiences; (2) Selection, where individuals choose an appropriate regulation strategy based on goals and situational factors; and (3) Implementation, which involves putting the selected strategy into action, varying in complexity depending on the approach. Different ER strategies are commonly categorized as adaptive (e.g., reappraisal, problem-solving) or maladaptive (e.g., suppression, rumination) based on their correlation with psychopathology [13]. However, the effectiveness of these ER strategies (in terms of achieving ER goals) is influenced by individual and contextual factors [12, 14, 15]. Difficulties regulating emotions according to one’s goals, along with the use of socially and contextually inappropriate strategies that address immediate coping needs but result in long-term maladaptive outcomes, are often referred to as emotion dysregulation (ED) [15]. Research consistently shows that autistic individuals typically experience greater ED than the general population [16, 17]. Evidence suggests a link between ED and the defining symptoms of autism [18, 19].

Bemmouna and Weiner [20] adopted Linehan’s biosocial theory [21] to conceptualize factors contributing to enhanced ED in autism. The model acknowledges the interplay between challenges related to the core features and biological vulnerabilities of ASD, as well as psychosocial environmental factors, such as the high risk of invalidating experiences arising from adverse childhood events, social rejection, and subsequent internalized stigma. While ED among autistic individuals is often evident from an early age [22], manifesting as outbursts and behavior problems, these issues can become more pronounced and impactful during adolescence.

Adolescence is the transitional phase from childhood to adulthood that prepares individuals for adult roles and responsibilities. It is marked by substantial physical, psychological, and social development [23]. Adolescence encompasses and extends beyond puberty—a period of biological maturation notable for physical growth and sexual development—and includes significant shifts in motivations, affective and cognitive functioning, and social interactions [24]. Adolescence is a key developmental period that shapes future health behaviors and life trajectories [25] and is associated with an increased risk of developing various mental health issues [26]. Although the definition of adolescence varies, Sawyer et al. [27] proposed a notable extended definition, recognizing physical maturation as the onset and the increasingly delayed timing of key social-role transitions as the endpoint, advocating for an age range of 10–24 years. This expanded definition may be particularly relevant for autistic adolescents since evidence suggests that daily living skills in young autistic individuals often lag behind age-level expectations [28, 29].

To date, five systematic reviews on ER in autism have been published. One of these specifically analyzed the available measures of ER and their relationship to different ER frameworks [30]. Cibralic et al. [22] focused their analysis on the nature of ER in young autistic children aged 12–72 months while referencing studies encompassing a broader age range. In comparison, another systematic review [31] examined ER within parent-mediated interventions for autistic children aged six years and younger, assessing if and how ER was measured and addressed, as well as its relationship with challenging behaviors. Most recently, two meta-analyses on ER in autistic individuals have been conducted. One focused on ED severity in autism compared to non-autistic populations across ages 1–63 years [32], while the other examined ER and psychological factors in children and adolescents with ASD, with a mean age of 2.2–18 years [33]. The substantial heterogeneity of age ranges in these reviews hampers the interpretation of results for specific age groups.

Considering evidence for age-related changes in ER within the general population [6] and an unfavorable shift in ER during adolescence [34], it is essential to adopt a developmental perspective when studying autism [35, 36]. This is particularly important in adolescence, a critical period when comorbidities are highly prevalent in both clinical [8] and population-based samples [37]. Recognized as a core transdiagnostic factor underlying many psychiatric and neurodevelopmental disorders [5, 38], understanding ER in autistic adolescents within the Research Domain Criteria (RDoC) framework [6] may inform the planning of targeted interventions [35, 39, 40].

## Study aims

This systematic review aims to synthesize current research on ER in autistic adolescents aged 10–24 years and address the following research questions: (1) What is our current understanding of ER in autistic adolescents aged 10–24 years? (2) What are the differences in ER between autistic and non-autistic adolescents?

## Methods

The present systematic review was preregistered with PROSPERO (CRD42024529184) and was conducted in accordance with the latest Preferred Reporting Items for Systematic Reviews and Meta-Analyses (PRISMA) guidelines [41]. See Additional File 2 for the PRISMA Checklist.

### Eligibility criteria

For the study population, autism diagnoses were required to be validated according to recognized criteria, specifically DSM-5 [42] or ICD-10 [43]. Consequently, self-reported autism diagnoses and samples based solely on autism questionnaires with established cut-off symptom scores were excluded. The age range of the study population was set to 10–24 years, consistent with recent definitions of extended adolescence [27]. We included only original, peer-reviewed journal articles published in English that presented quantitative and/or qualitative measures of ER in autistic adolescents. Additionally, we included only studies that reported data on a specific subgroup or the total group within our defined age range. Studies addressing broader concepts (e.g., behavioral problems, behavioral regulation, aberrant behaviors, and challenging behavior) were screened for potential relevance to the construct of emotion regulation.

### Search strategy

To develop a structured search strategy, we consulted a senior librarian and conducted an electronic search across MEDLINE, PsycINFO, Web of Science, and Scopus. We made minor adaptations to the search string for each database. Table 1 presents the search strategies used in the OVID databases. The initial searches were carried out on May 28, 2024, and database alerts were established to monitor subsequently published studies. The final update was performed on May 06, 2025. Additionally, we manually searched websites and conducted backward citation searches of the reference lists of included studies and relevant systematic reviews on the topic.

**Table 1:** Search strategy

Ovid Databases (APA PsycInfo, MEDLINE ALL)	
1	(Autis* or Asperger* Syndrome or Pervasive Development* Disorder* or ASD or ASC or Neurodevelopment* Disorder*).mp [mp = title, original title, abstract, name of substance word, subject heading word, unique identifier]
2	((emotion* or affect* or mood) and (co-regulation or regulation or dysregulation)).mp [mp = title, original title, abstract, name of substance word, subject heading word, unique identifier]
3	(adolescen* or teenage* or young or juvenile or young adult*).mp [mp = title, original title, abstract, name of substance word, subject heading word, unique identifier]
4	1 and 2 and 3
5	remove duplicates from 4

### Study selection and data collection

We imported all identified references into Rayyan [44] and deduplicated. The first (JM) and last author (JB) independently screened titles and abstracts for eligibility, and subsequently reviewed retrieved full texts to assess inclusion criteria, again using Rayyan. Any disagreements were resolved through discussion involving a third reviewer (HZ). JM and JB independently extracted data from the included full texts, followed by mutual cross-checking; all data where then reviewed by HZ, with disagreements resolved by consensus. Extracted data items included: study characteristics (first author, year, country of origin, type of study, and study design), sample description, recruitment method, study aims, measures of ER and informants, additional relevant measures, key results and psychometric properties of ER measures, and, for qualitative studies, relevant themes and subthemes with illustrative examples. To address developmental heterogeneity, we specifically extracted information on participant age ranges, and, where possible categorized findings into adolescent subgroups of (10–14 years), middle (15–19 years), and late adolescence/young adulthood (20–24 years) [44]).

The RDoC framework provides a dimensional approach to investigating mental health disorders by integrating multiple levels of information–biological, psychological, and environmental–to better understand their complexity [45]. This integrative approach enables researchers to focus on the mechanisms underlying emotional and behavioral functioning. In this systematic review, we applied the RDoC framework to examine ER in autistic adolescents across four RDoC units of analysis: Circuitry, Physiology, Behavior, and Self-/Other Report Questionnaires.

### Quality assessment

Quality assessment of the selected studies was conducted independently by authors JM and JB using the Mixed Methods Appraisal Tool (MMAT) [46]. The MMAT is designed to evaluate the methodological quality of qualitative, quantitative randomized controlled trials, quantitative non-randomized trials, quantitative descriptive studies and mixed methods studies within systematic reviews. It employs distinct sets of five quality criteria tailored to assess each specific study designs. All discrepancies were resolved through discussion and consensus involving the third author, HZ. All studies, irrespective of their methodological quality outcomes, underwent data extraction and synthesis.

### Data synthesis

This review adopted a convergent integrated approach, as recommended for mixed-methods reviews that integrate quantitative and qualitative data [47]. Quantitative data were ‘qualitised’, meaning statistical outcomes were translated into descriptive themes to facilitate their integration with qualitative findings [47]. Thematic synthesis [48] was employed to address expected heterogeneity in study designs and outcomes, categorizing the extracted data into themes and subthemes based on shared meanings. Synthesis involved three steps: (1) line-by-line coding of findings, (2) development of descriptive themes and organization of codes into these themes, and (3) generation of analytic themes. One reviewer initially coded all extracted data, which was then discussed and refined with a second reviewer until consensus was reached. Results are organized by theme, with quantitative and qualitative findings presented together. When studies provided age-stratified or subgroup analyses, we synthesized these results by developmental stage.

## Results

### Study selection

We identified 3979 articles across databases after removing 727 duplicates. The inter-rater reliability for title and abstract screening showed moderate agreement (*k* = 0.647; 95% CI, 0.629–0.666). Full-text assessment was conducted on 186 articles, with 159 ultimately excluded. Through backward citation searching and website screening, we identified six additional articles, five of which were included in the final analysis. The inter-rater reliability for full-text analysis was strong (*k* = 0.842; 95% CI, 0.785–0.899).

**Fig. 1:**
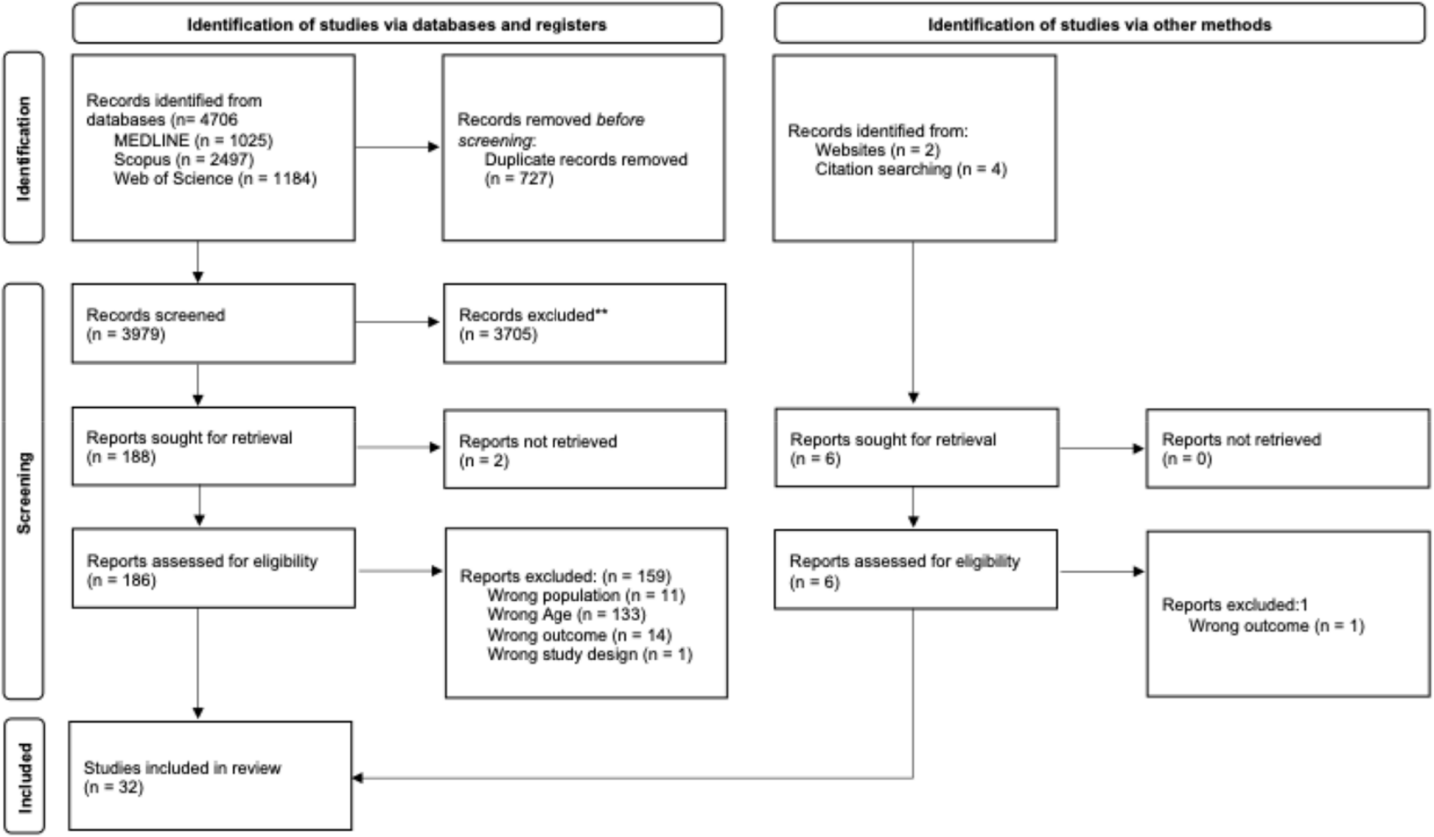
PRISMA 2020 flow diagram

Finally, 32 studies met all eligibility criteria and were included in the systematic review. The PRISMA flowchart is presented in Fig. 1.

### Methodological quality of included studies

The two qualitative studies met all five MMAT quality criteria. Among the quantitative studies, the majority (*N =* 25) fulfilled at least three quality criteria; two studies met only two, and one study met only one criterion. Common methodological limitations in quantitative studies included a high risk of non-response bias (*N =* 16) and lack of a representative ASD sample (*N =* 11). Additional File 1 details the quality assessment of all included studies according to the MMAT [46].

### Study characteristics

Detailed study characteristics are summarized in Table 2. The included studies were published between 2013 and 2024 and originated from 12 countries. The majority (*N =* 15) were from the United States. Other contributions included three studies from the United Kingdom, three from Australia, two from Canada, two from Turkey, and one each from Hong Kong, Taiwan, Italy, Spain, Israel, the Republic of Korea, and Ireland. Quantitative study sample sizes ranged from 7–722 participants. The two qualitative studies included 13 adolescent participants and nine informants (parents and various professionals) representing autistic adolescents. In total, the studies involved 2,791 participants, of whom 2,080 were diagnosed with ASD. Two studies did not report gender distribution; in the remaining studies, 57.4% of all participants were male, and 61.9% of the autistic subsample were male.

**Table 2:** Detailed characteristics of included studies

Author (year)	Country	N of participants (*N =* sex/gender)	Mean age in years (SD), range	Mean IQ (SD), range	ER measure (Informants)	Findings regarding emotion regulation (ER)
[49] Brewe et al. (2021)_1_	USA	ASD group:* N =* 37 (29 M, 8 F)	15.28 (2.21), 12–21	103.31 (17.13)	EDI (caregivers)	Therapeutic alliance was associated with improvements in the dysphoria subscale at post-treatment but not with the reactivity subscale. Internal consistencies for both subscales ranged from .83 to .95 at various time points. [Implementation]
[50] Charlton et al. (2020)_2_	USA	ASD group:* N =* 27 (20 M, 7 F)	18.54 (2.02), 16–24	103.88 (10.67)	RSQ_a_ (adolescents, caregivers)	Individuals with comorbid anxiety or mood disorders reported greater involuntary engagement (IE) and lower voluntary engagement (VE) than those without these disorders. However, caregiver reports showed no differences in IE or VE between individuals with and without anxiety or mood disorders. Moreover, IE was linked to internalizing and externalizing symptomatology only in caregiver ratings. Internal consistency was .86 for the VE subscale and .83 for the IE subscale. [Selection and Implementation]
[51] Chiu et al. (2024)_2_	Hong Kong	ASD group: *N =* 23 (16 M, 7 F); non-ASD group: *N =* 32 (8 M, 24 F)	ASD group: 15.78 (1.78), 13–18; non-ASD group: 16.38 (1.34), 13–18	ASD group: 90.30 (18.11); non-ASD group: 116.34 (12.79)	EDI (parents/ caregivers) HRV	The ASD group exhibited greater emotion dysregulation than the non-ASD group on both the reactivity and the dysphoria subscales; however, no significant differences in HRV were found between the groups. In the ASD group, higher levels of reactivity and dysphoria were both associated with lower HRV. Internal consistency was .98 for the reactivity subscale and .84 for the dysphoria subscale. [Implementation]
[52] Chou et al. (2023)_1_	Taiwan	Experimental ASD group: *N =* 28 (23 M, 5 F); control ASD group: *N =* 38 (29 M, 9 F)	All: 13.93 (0.93); experimental group: 13.89 (0.87); control group: 13.97 (0.99)	n/a	ERS (adolescents, teachers)	Adolescents in the experimental group showed improvement over time compared to the waitlist control group in teacher-reported overall ER. These findings were also evident at the subscale level of the ERS. Self-reports indicated similar trends, without reaching statistical significance. Internal consistencies of the ERS ranged from .92. to .95 [Identification and Selection]
[53] Coffman et al. (2024)_1_	USA	ASD group: *N =* 15 (12 M, 3 F)	15.62 (1.54), 13.14—18.32	n/a	ABC-2-I, BRIEF-2, EDI (parents)	The ASD group showed improvement over time on the reactivity subscale of the EDI, the irritability subscale of the ABC-2 and in the emotion regulation index of the BRIEF-2. Additionally, post-hoc analyses revealed relevant improvements in these measures, as well as in the dysphoria subscale of the EDI, from the start of the intervention to multiple post-intervention assessments. No information on internal consistencies. [Implementation]
[54] Conner et al. (2023)_2_	USA	ASD group:* N =* 57 (46 M, 11 F)	18.56 (2.04), 16–24	105.82 (12.40)	DERS (adolescents)	Associations were found between ER, ASD traits, anxiety, and depression. In a regression model, ER accounted for a high percentage of the variance in depression and anxiety. Internal consistencies of DERS subscales ranged from .79 to .97. [Identification, Selection and Implementation]
[55] Conner et al. (2022)_2_	USA	ASD group:* N =* 78 (61 M, 15 F, 2 O)	14.87 (2.27), 12–20	102.44 (16.23)	EDI (caregivers)	Associations were found between the reactivity subscale and the dysphoria subscale with intolerance of uncertainty. Both EDI subscales mediated the association between intolerance of uncertainty, anxiety, and depression. Internal consistency was .93 for the reactivity subscale and .86 for the dysphoria subscale. [Implementation]
[56] Conner et al. (2019)_1_	USA	ASD group:* N =* 17 (15 M, 2 F)	14.94 (1.54), no age-range reported	98.47 (11.71)	EDI, ABC-I (parents), RSQ_b_ (adolescents, parents)	Adolescents showed parent-reported improvements across various ER measures. Parent-reports on the RSQ indicated improved ER whereas self-reports did not. Internal consistency was .94 for the EDI and .85 for the ABC-I. RSQ internal consistencies ranged from .55 and .82 in the parent version and from .82 and .92 in the self-report version. [Selection and Implementation]
[57] Dağdelen et al. (2021)_2_	Turkey	ASD group: *N =* 60 (30 M, 30 F); ADHD group: *N =* 60 (30 M, 30 F); control group: *N =* 60 (30 M, 30 F)	ASD group: 14.02 (1.44); ADHD group: 14.00 (1.43); control group: 13.55 (1.41), no age-range reported	ASD group: 95.47 (10.31); ADHD group: 95.47 (10.31); control group: 94.45 (10.82)	DERS (adolescents)	The ASD group had higher emotion dysregulation scores compared to both the ADHD group and the control group. Additionally, emotion dysregulation was associated with lower theory of mind scores. Internal consistency data were not reported. [Identification, Selection and Implementation]
[58] Fatta et al. (2024)_1_	Italy	Experimental ASD group: *N =* 18 (12 M, 6 F); waitlist ASD group: *N =* 19 (14 M, 5 F)	Experimental ASD group: 15.14 (2.26), 12.3–18.2; waitlist ASD group: 15.5 (1.74), 12.2–18.2	Experimental ASD group: 109.65 (10.51); waitlist ASD group: 106.43 (16.22)	BRIEF-2 (parents)	A group by time interaction effect was found for the BRIEF-2 emotion regulation index, indicating a relevant improvement in ER abilities in the experimental group. These improvements persisted at follow-up. Internal consistency was .90 for the emotion regulation index. [Implementation]
[59] Gerber, et al. (2024)_1_	USA	Experimental ASD group: *N =* 20 (16 M, 4 F); control ASD group:* N =* 20 (16 M, 4 F)	Experimental ASD group: 14.27 (1.76); control ASD group: 14.17 (1.41), no age-range reported	Experimental ASD group: 103.47 (16.93); control ASD group: 107.47 (14.40)	BRIEF-2 (adolescents, parents)	A group effect was found for the parent-reported BRIEF-2 emotion regulation index at the 3-month follow-up, whereas adolescent self-reports indicated marginal improvements Internal consistency ranged from .89 to .91 for the parent-report and from .85 to .88 for the adolescent-report. [Implementation]
[60] Goldfarb et al. (2021)_3_	Israel	ASD group:* N =* 10 (5 M, 5 F); *N =* 6 (3 M, 3 F) within the age-range 10–24 y	Age range of included adolescents: 18–24	n/a	In-depth interview, thematic analysis (adolescents)	Adolescents reported using self-harming behaviors to regulate their emotions, such as scratching with wire to shift away from emotional pain, self-hitting to prevent aggression towards others during anger and severe anxiety, and self-hitting to maintain self-control or regain focus in emotionally challenging situations. [Selection and Implementation]
[61] Gormley et al. (2022)_2_	Ireland	ASD group:* N =* 43 (32 M, 11 F)	13.1 (2.5), no age-range reported	IQ > 70	ERICA (adolescents,); ERC (parents)	Adolescents exhibited more ER difficulties relative to normative data. A negative association was found between self-reported ER abilities and alexithymia. Regression analysis indicated that alexithymia accounted for a substantial proportion of variance in ER abilities. Parent reported emotional lability and negativity were strongly linked to ASD severity. Internal consistency was .84 for ERICA and ranged from .73 to .86 for ERC subscales. [Identification, Selection and Implementation]
[62] Khor et al. (2014)_2_	Australia	ASD group *N =* 31 (26 M, 5 F)	14.46 (1.83), 12–18	99.87 (14.33), 70–130	RSQ (adolescents, parents)	The self-reports and parent-reports differed for most subscales, except for similar levels of reported disengagement coping and involuntary disengagement. The use of maladaptive ER strategies was associated with both self- and parent-reported behavioral and emotional problems. Internal consistencies for subscales and both respondents ranged from .64 to .89. [Selection and Implementation]
[63] Kim et al. (2021)_1_	Republic of Korea	ASD group:* N =* 7 (7 M)	20.29 (1.11), 19–22	92.57 (n/a), 76–120	K-BDEFS (adolescents, caregivers)	Self-reports showed improvements on the regulation of emotions subscale from pre- to post-intervention. In contrast, caregiver-reports indicated a decline in scores on the same subscale during the same period. Internal consistency data were not reported
[64] Kose et al. (2023)_2_	Turkey	ASD group: *N =* 50 (38 M, 12 F); non-ASD group: *N =* 73 (35 M, 38 F)	ASD group: 14.1 (2.2), 11–18; non-ASD group: 14.6 (2.6), 11–18	ASD group: 98.02 (15.24)	ERQ (adolescents)	There were no differences in the ER subscales of cognitive reappraisal and expressive suppression between the two groups, nor was there a gender effect. High use of cognitive reappraisal was associated with better social skills and social functioning in the ASD group. Internal consistency was .78 for cognitive reappraisal and .73 for expressive suppression. [Selection]
[65] López-Pérez et al. (2018)_2_	Spain	ASD group: *N =* 30 (30 M); non-ASD group: *N =* 30 (30 M)	ASD group: 10.97 (0.88), 10–12; non-ASD group: 11.07 (0.86), 10–12	ASD group: 101.50 (11.98); non-ASD group: 103.80 (10.71)	DERS (adolescents)	Group differences were observed, including an interaction between emotion dysregulation and group. Post-hoc analyses revealed greater difficulties in the ASD group regarding goal-directed behavior, emotional awareness, and emotional clarity, whereas no differences emerged in non-acceptance, impulse control, or regulation strategies. Internal consistency for subscales ranged from .71 to .78. [Identification, Selection and Implementation]
[66] Mazefsky et al. (2014)_2_	USA	ASD group: *N =* 25 (24 M, 1 F); non-ASD group: *N =* 23 (22 M, 1 F)	ASD group: 15.22 (2.25), 12–19; non-ASD group: 15.56 (2.76), 12–19	ASD group: 110.48 (13.59); non-ASD group: 113.23 (12.87)	RSQ (adolescents)	No correlations were found between age, pubertal development, ASD traits and RSQ subscales. The ASD group reported greater use of both voluntary and involuntary disengagement strategies, as well as involuntary engagement strategies, compared to the non-ASD group. Distinct patterns of correlations among ER strategies were observed, with various strategies linked to both self- and parent-reported psychopathology. Internal consistency for the RSQ was .95 in the ASD group. [Selection and Implementation]
[67] Mazefsky et al. (2020)_2_	USA	ASD group: *N =* 25 (24 M, 1 F); non-ASD group: *N =* 23 (22 M, 1 F)	ASD group: 14.95 (2.47), 12–19; non-ASD group: 15.5 (2.76), 12–19	ASD group: 115.00 (14.60); non-ASD group: 113.23 (12.87)	CBCL-EDI (parents) fMRI	Brain activity in specific regions (left and right insula, right pulvinar, left dorsolateral cortex) predicted emotion dysregulation scores in the ASD group. Prolonged activity in the prefrontal cortex and salience network in response to negative words was observed. Internal consistency for the CBCL-EDI was .87. [Implementation]
[68] Phillips et al. (2024)_1_	UK	ASD group: *N =* 27 (27 F); Non-ASD group: *N =* 215 (7 M, 208 F) Both groups with symptoms of BPD	All: 16.48 (0.98), 13–17	No intellectual disability in ASD group	DERS (adolescents)	The ASD group showed a reduction in emotion dysregulation scores from pre- to post intervention in an exploratory service evaluation. There were no differences in change scores between the ASD group and the non-ASD group. Internal consistency of the DERS ranged from .88 to .96. [Identification, Selection and Implementation]
[69] Pickard et al. (2020)_2_	UK	ASD group: *N =* 61 (42 M, 19 F); non-ASD group: *N =* 62 (26 M, 36 F)	ASD group: 13.46 (1.77), 11–17; non-ASD group: 13.52 (1.57), 11–17	ASD group: 98.16 (13.99); non-ASD group: 100.76 (11.55)	CERQ (adolescents)	There were no differences between the ASD group and the non-ASD group regarding adaptive and maladaptive ER scores. An association between maladaptive ER scores and social anxiety was found in the ASD group. Internal consistency for the CERQ ranged from .84 to .89 in the ASD group. [Selection]
[70] Rosenthal et al. (2013)_2_	USA	ASD group 11–13 years: *N =* 50 (38 M, 12 F); ASD group 14–18 years: *N =* 36 (31 M, 5 F)	ASD group 11-13y.: n/a (n/a) ASD group 14-18y.: n/a (n/a)	ASD group 11-13y.: 109.38 (19.07); ASD group 14-18y.: 104.58 (17.16)	BRIEF (caregivers)	Both groups exhibited clinically elevated scores (T > 65) on the shift subscale. Although T-scores on the emotional control subscale were also elevated, they did not reach clinical relevance. Post-hoc testing revealed no differences across the age groups. Internal consistency data were not reported. [Implementation]
[71] Russel et al. (2024)_1_	USA	ASD group: *N =* 12 (7 M, 5 F);	14.25 (1.22), 11–16;	97.42 (19.0);	EDI (caregivers)	Post-hoc analysis revealed an increase of ER skills following the intervention. This improvement did not persist at the 10-week follow-up. Internal consistency data were not reported. [Implementation]
[72] Salem-Guirgis et al. (2019)_1_	Canada	ASD group:* N =* 23 (19 M, 4 F)	15.65 (2.57), 12–23	103.96 (12.98)	RRS, ERQ-CA (adolescents); ERC (parents)	Post-intervention, adolescents reported improvements in the RRS reflection subscale, which were maintained at the 10-week follow-up. Self-reported effects were observed on the ERQ-CA cognitive reappraisal subscale at the 1-week follow-up, and parents reported effects on the ERC emotion regulation subscale at 10-week follow-up. Internal consistency ranged from .81 to .90 for the RRS, .84 to .85 for the ERQ-CA and .75 to .85 for the ERC. [Selection and Implementation stages]
[73] Santomauro et al. (2016)_1_	Australia	Experimental ASD group: *N =* 10 (n/a); waitlist ASD group: *N =* 10 (n/a)	Experimental ASD group: 16.00 (1.33); waitlist ASD group: 15.50 (1.43), no age-range reported	Verbal IQ ≥ 85	ERQ (adolescents)	No group differences were found on the cognitive reappraisal and suppression subscales. A time effect was observed for cognitive reappraisal with post-hoc tests indicating changes from baseline to post-intervention, and from post-intervention to 3-month follow up. Better theory of mind correlated with higher cognitive reappraisal scores. Internal consistency was .86 for cognitive reappraisal and .72 for suppression. [Selection]
[74] Santomauro et al. (2017)_3_	Australia	ASD group: *N =* 7 (6 M, 1 F); ASD group represented by parents:* N =* 9 (9 M);	ASD group represented by parents: 15.43 (1.99), 13–19; ASD group: 20.71 (3.09), 14–23	n/a	Focus groups and interviews (adolescents, parents, different professional groups)	The ASD group, caregivers and professional groups highlighted that ER difficulties significantly impact autistic adolescents’ lives. Key themes identified were: (1) triggers of distressing emotions (e.g. sensory sensitivities), (2) difficulties with emotional awareness (e.g. alexithymia and the need for external support), (3) ER strategies (both adaptive and dysfunctional strategies) and (4) consequences of distressing emotions (e.g. academic and social challenges). [Identification, Selection and Implementation]
[75] Shaffer et al. (2023)_1_	USA	ASD group: *N =* 19 (n/a)	14.80 (1.38), 13–18	96 (18.51), not specified by group (child and adolescents)	EDI, ABC-2-I, BRIEF-2 (caregivers)	The intervention showed an effect on the reactivity subscale, but not on the dysphoria subscale of the EDI. On the irritability subscale of the ABC-2, changes were observed across all time points. Additionally, the emotion regulation index of the BRIEF-2 indicated changes, both post-treatment and at the follow-up assessments. No information on internal consistencies. [Implementation]
[76] Skwerer et al. (2019)_2_	USA	ASD group: *N =* 32 (22 M, 10 F)	14.79 (1.9), 12–18	NVIQ: 48.97(12.97)	EDI (caregivers)	Emotion dysregulation was not related to ASD traits, age group, NVIQ, or gender. Both reactivity and dysphoria scores were associated with the number of comorbid symptoms, while only dysphoria linked to generalized anxiety. Adaptive functioning scores did not correlate with EDI subscales; however, reactivity correlated with externalizing behaviors, while dysphoria showed no association with internalizing behaviors. In regression analysis, a composite of external and internal behaviors was predicted by the number of comorbid symptoms, but not by EDI scores. Internal consistency data were not reported. [Implementation]
[77] Timko et al. (2021)_2_	USA	ASD group: *N =* 29 (29 F) Anorexia nervosa (AN) group: *N =* 22 (22 F)	ASD group: 12.30 (2.25), 10–17; AN group: 14.81 (2.33), 10–18	ASD group: 96.3 (24.69), 42–145 AN group: 105.84 (13.95), 83–142	BRIEF (caregivers)	The ASD group’s mean score on the shift subscale was in the clinical range (T > 65) compared to norms. A higher proportion of the ASD group scored in the clinical range on both the shift and emotional control subscales than the AN group. In the ASD group, the shift score predicted restricted repetitive behaviors in a linear regression analysis. Internal consistency data were not reported. [Implementation]
[78] Wieckowski et al. (2020)_2_	USA	ASD group:* N =* 722 (576 M, 146 F)	12.95 (3.41), 4–20	NVIQ: 75.11 (28.23)	EDI (caregivers)	Autistic adolescents in specialized inpatient psychiatric units exhibited clinically significant emotion dysregulation relative to general population norms. Among those under age 13, no gender differences were observed in EDI scores; however, females aged 13 and older scored higher on the dysphoria subscale than males. Furthermore, being verbal correlated with higher dysphoria scores on the EDI. Internal consistency data were not reported. [Implementation]
[79] Woodcock et al. (2020)_2_	UK	ASD group:* N =* 20 (16 M, 4 F); non-ASD group: *N =* 80 (64 M, 16 F)	ASD group: 13.3 (n/a), 11–17; non-ASD group: 13.3 (n/a), 10–17	ASD group: 107.7 (18.1); non-ASD group: n/a	CAMS, CSMS (adolescents); BRIEF (parents)	In the ASD group, no correlations were found between self-reported anger and sadness regulation scores and decisions as proposers or responders in the ultimatum game. Average T-scores on the shift and emotional control subscales of the BRIEF were clinically elevated (> 65) relative to norms. Poorer emotional control was linked to lower acceptance rates of unfair offers in the ultimatum game. No information on internal consistencies. [Implementation]
[80] Yager et al. (2013)_2_	Canada	ASD group* N =* 22 (19 M, 3 F); non-ASD group; *N =* 22 (19 M, 3 F)	ASD group: 14.17 (2.25), 11–18; non-ASD group: 14.12 (2.27), 11–18	ASD group: 101.05 (10.53); non-ASD group: 100.18 (10.60)	MSCS (caregivers)	There was an association between the ER subscale of the MSCS and ASD traits. The ASD group exhibited lower scores in the ER subscale compared to the non-ASD group. Internal consistency was .89 for the ER subscale. [Implementation]

*ABC-I* Aberrant Behavior Checklist—Irritability subscale, *ABC-2-I* Aberrant Behavior Checklist—Second Edition—Irritability subscale, *ADHD* Attention Deficit Hyperactivity Disorder, *ASD* Autism Spectrum Disorder, *BRIEF* Behavior Rating Inventory of Executive Function, specifically the subscales shift and emotional control, *BRIEF-2* Behavior Rating Inventory of Executive Function—Second Edition, specifically the Emotional Regulation Index, *BPD* Borderline personality disorder, *CAMS* Children’s Anger Management Scale, *CBCL-EDI* The Child Behavior Checklist—Emotion Dysregulation Index, *CERQ* Cognitive Emotion Regulation Questionnaire, *CSMS* Children’s Sadness Management Scale, *DERS* Difficulties in Emotion Regulation Scale, *EDI* Emotion Dysregulation Inventory, *ERC* Emotion Regulation Checklist, *ERICA* Emotion Regulation Index for Children and Adolescents, *ERQ* Emotion Regulation Questionnaire, *ERQ-CA* Emotion Regulation Questionnaire for Children and Adolescents, *ERS* Emotion Regulation Scale, *fMRI* functional magnetic resonance imaging, *F* Female, *HRV* resting heart rate variability, *ID* Intellectual Disabilities, *K-BDEFS *Korean Version of Barkley Deficits in Executive Functioning Scale, specifically the factor *regulation of emotion*, *M* Male, *MSCS* Multidimensional Social Competence Scale, *NVIQ* Nonverbal IQ, *Non-ASD* Non-autistic adolescents, refers to adolescents with no history of neurological or psychological condition, if not otherwise stated, *O* other genders (including transgender, non-binary and agender), *RRS* Ruminative Response Scale, *RSQ* Response to Stress Questionnaire – Social stress version (RSQ_a_ only subscales involuntary engagement and voluntary engagement, RSQ_b_ only subscales involuntary engagement and involuntary disengagement). Information in square brackets in the 'Findings regarding ER' column indicates the stage(s) of the extended process model of emotion regulation to which the results predominantly pertain

_1_Interventional study; _2_Cross-sectional study; _3_Qualitative study

Of the included studies, two provided samples with participant ages falling entirely within the early adolescent range (10–14 years), while none focused exclusively on the middle (15–19 years) or late adolescents/young adult (20–24 years) ranges. Sixteen studies included participants spanning both early and middle adolescence (with ages between 10–19 years), three included both middle and late adolescence (15–24 years), and five studies covered the full span from early to late adolescence (10–24 years). Six studies did not clearly report a participant age range.

Only a few studies specifically analyzed age-related differences: one compared younger and older adolescent subgroups [70], one used dichotomized age in regression analyses [78], one performed group comparisons by age [76], and two examined age as a continuous variable in correlation analyses [64, 66]. However, most studies included wide age ranges without conducting age-stratified analyses, limiting developmental interpretation. Overall, most research has employed broad age ranges with limited attention to discrete developmental subgroups. Additional File 3 provides a matrix visualization of participant age coverage, enabling visual comparison of developmental stages represented and illustrating how study findings correspond to specific age groups.

### Study design

Most included studies were quantitative (*N =* 30), comprising a total of 18 cross-sectional and 12 interventional studies. Eight cross-sectional studies [50, 54, 55, 61, 62, 70, 76, 78] focused on ER solely in autistic adolescent populations without investigating comparison groups. An additional 10 cross-sectional studies [51, 57, 64–67, 69, 77, 79, 80] compared ER measures in autistic and non-autistic adolescent populations. Only two studies explicitly focused on participants with below-average cognitive functioning [76, 78].

Seven out of the 12 interventional studies reported outcomes of programs primarily focused on enhancing ER capabilities in autistic adolescents [49, 52, 53, 56, 68, 72, 75], while the other five studies addressed internalizing symptoms [59, 73], executive functioning and adaptive skills [63], and social skills [58, 71], with ER as a secondary outcome. A total of 11 interventional studies, including three randomized controlled trials (RCTs) [49, 52, 53, 56, 58, 59, 63, 71–73, 75], investigated the effects of various interventions on ER capabilities in autistic adolescents without a non-autistic comparison group. One interventional study compared outcomes of a non-adapted Dialectical Behavioral Therapy (DBT) program for two groups: autistic adolescents with co-occurring self-harming behavior and emerging borderline personality disorder symptoms, and non-autistic adolescents with similar co-occurring traits [68].

Additionally, we identified two qualitative studies: one employed focus groups with a diverse range of caregivers and conducted in-depth interviews with autistic adolescents [74], while the other involved semi-structured in-depth interviews with a larger number of participants across diverse age ranges; however, we focused exclusively on the adolescent participants [60]. Both qualitative studies utilized thematic analysis.

### Instruments

The most frequently used quantitative measure of ER was the parent-reported Emotion Dysregulation Inventory (EDI) [81], utilized in nine studies and primarily assessing the implementation stage of the extended process model [12]. The Response to Stress Questionnaire – Social Stress Version (RSQ) [82] and Difficulties in Emotion Regulation Scale (DERS) [83] were each used in four studies: the RSQ, although with varying combinations of its subscales. The RSQ as either parent- or self-report, focused mainly on the selection and partially the implementation stage, while the self-report DERS spanned identification, selection, and implementation. The Behavior Rating Inventory of Executive Function (BRIEF/BRIEF-2) [84, 85] focusing on the Emotional Regulation Index was used in seven studies (mostly as parent report), and the Aberrant Behavior Checklist – Irritability subscale (ABC-I /ABC2-I) [86, 87] in three, both targeting dysregulation outcomes (implementation). Other ER tools, including the Emotion Regulation Questionnaire (ERQ) [88], and a variety of less commonly used scales, varied in their focus and informant. The diversity of tools and informants contributed to heterogeneity in findings and limited comparability between studies. Table 3 summarizes all ER measures used, their main informants, and the extended process model stages they primarily assess.

**Table 3:** Emotion regulation measures mapped onto stages of the Extended Process Model (EPM)

Instrument	EPM – Stages			Studies (N)
	Identification	Selection	Implementation	
^1^Emotion Dysregulation Inventory (EDI) [81]			x	9
^1^Child Behavior Checklist, Emotion Dysregulation Index (CBCL-EDI) [19]			x	1
^2^Difficulties in Emotion Regulation Scale (DERS) [89]	x	x	x	4
^1/2^Response to Stress Questionnaire (RSQ) [82]		x	x	4_a_
^1^Behavior Rating Inventory of Executive Function, 1st & 2nd Ed. (BRIEF-2) – Emotional Regulation Index (ERI) [84, 85]			x	7_b_
^1^Aberrant Behavior Checklist – 1st & 2nd ed. – Irritability subscale (ABC2-I) [86, 87]			x	3
^2^Emotion Regulation Questionnaire (ERQ) [88]		x		2
^2^Emotion Regulation Questionnaire for Children and Adolescents (ERQ-CA) [90]		x		1
^2^Emotion Regulation Scale (ERS) [52]	x	x		1
^2^Emotion Regulation Index for Children and Adolescents (ERICA) [91]	x	x	x	1
^1^Emotion Regulation Checklist (ERC) [92]			x	1
^1/2^Korean Version of Barkley Deficits in Executive Functioning Scale – subscale regulation of emotion (K-BDEFS) [93]			x	1
^2^Ruminative Response Scale (RRS) [94]		x		1
^2^Cognitive Emotion Regulation Questionnaire (CERQ) [95]		x		1
^2^Children’s Anger Management Scale (CAMS) [96]		x	x	1
^2^Children’s Sadness Management Scale (CSMS) [97]		x	x	1
^1^Multidimensional Social Competence Scale – subscale emotion regulation (MSCS) [98]			x	1

_a_RSQ was utilized in three ways: Charlton et al. [50] used only *involuntary engagement* and *voluntary engagement* subscales; Conner et al. [56] used only *involuntary engagement* and *involuntary disengagement* subscales; Mazefsky et al. [66] and Khor et al. [62] used all RSQ—Social Stress subscales

_b_BRIEF-2 used as both self- and parent-report by Gerber et al. [59]

^1^Paren/Caregiver-report, ^2^Self-report

Informant discrepancies were notable: across studies using the same instrument for parents and adolescents (e.g., RSQ, BRIEF-2), parent-reports indicated greater ER difficulties [50, 52, 56, 59, 62, 63] and sometimes stronger intervention effects [52, 56, 59]. In contrast, Kim et al. [63] found parents reported a decline in ER abilities, while adolescents reported improvement. Some studies found weak or no correlation between ER scores when different scales were used for parents and adolescents [61, 79], or did not examine correlations between different ER scales for both informants [72]. Additionally, Khor et al. [62] uniquely combined a self-report questionnaire with Ecological Momentary Assessment, illustrating methodological diversity.

### RDoC

Regarding the RDoC domains, we identified one study that examined ‘neural circuitry’ and included a parent-report questionnaire assessing ER difficulties. This study utilized functional magnetic resonance imaging (fMRI) during event-related trials that alternated between non-emotional and emotional stimuli to predict parent-reported ER difficulties using a regression model [67]. Another study investigated ‘physiology’ by examining resting heart rate variability (HRV) and its relationship to parent-reported ER difficulties [51]. None of the included studies focused on the ‘behavior’ domain. All other studies assessed ER through self-report, parent/caregiver-report, or both (see Table 2 for ER measures used in each study and Table 3 for instrument details).

### Main results

**Fig. 2:**
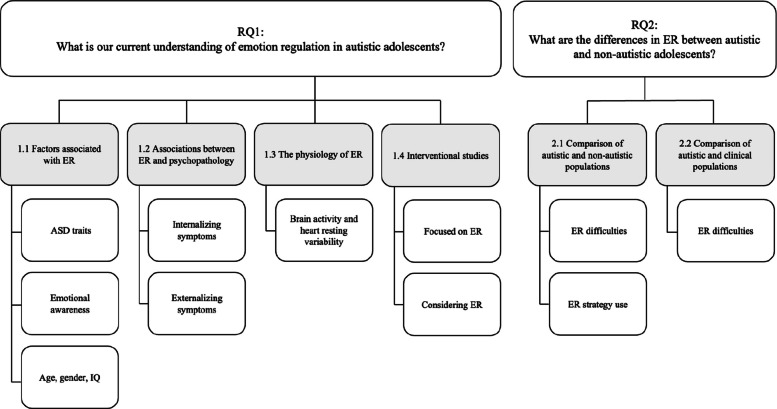
Thematic map

Following data transformation and thematic synthesis, we identified six main themes (see Fig. 2 for the thematic map). Four of these themes pertain to our first review question regarding our current understanding of ER in autistic adolescents: (1.1) Factors associated with ER, (1.2) associations between ER and psychopathology, (1.3) the physiology of ER, and (1.4) interventions targeting ER. Two themes address the second research question: (2.1) Autistic populations compared to non-autistic populations, and (2.2) autistic populations compared to clinical populations.

The following sections provide a narrative synthesis of qualitative and quantitative findings organized according to our research questions and thematic synthesis (see Table 2 for ‘qualitized’ result summaries and Additional File 3 for the detailed age range mapping across study results organized by themes).

#### Factors associated with ER

Five quantitative studies examined associations between age and ER: one compared participants aged 11–13 versus 14–18 years [70]; one used a < 13 versus ≥ 13 years grouping in regression analysis [78]; one compared 12–18 to 5–11 year-olds [76]; and two analyzed age as a continuous variable in samples ranging from 11–19 years [64, 66]. None found a direct association between age and ER. However, Wieckowski et al. [78] observed a small moderating effect of age on the gender–ER relationship in a minimally verbal outpatient sample.

For gender, two studies [64, 76] reported no ER differences in ER. These same studies also examined IQ in relation to ER. Skwerer et al. [76] used the Leiter-R for nonverbal IQ in a minimally verbal sample, while Kose et al. [64] employed the WISC-R for full-scale IQ in a group with average cognitive ability; both found no association between IQ and ER.

Nine studies explored associations between ASD traits— such as core ASD symptom severity, theory of mind (ToM), social skills, and sensory sensitivities—and ER [54, 57, 61, 64, 66, 73, 74, 76, 80]. Greater symptom severity was linked to increased ER difficulties in samples with average cognitive ability [54, 61, 80], but not in two studies: one with below-average cognitive ability [76] and one with average ability [66]. Higher ToM was linked to more adaptive ER [73], while lower ToM was associated with greater ER difficulties in a sample with an even gender distribution [57]. Social skills were positively associated with adaptive ER [64]. Qualitative findings indicated that both adolescents and parents identified social challenges and sensory sensitivities as triggers for distressing emotions that impair ER [74]; both groups also cited alexithymia as a relevant factor. Consistently, higher alexithymia was associated with lower ER abilities [61]. López-Pérez et al. [65] further found that young autistic male adolescents (10–12 years) experienced greater difficulties in emotional awareness and clarity than non-autistic peers.

#### Associations between ER and psychopathology

The use of maladaptive ER strategies—such as rumination, numbing, and self-blame—as well as difficulties with reactivity and dysphoria, were consistently linked to internalizing symptoms in six quantitative [50, 54, 55, 62, 69, 76] and two qualitative studies [60, 74], across the full adolescent age range and varying cognitive abilities. Similarly, maladaptive ER strategies and problems with reactivity and attention shifting were associated with externalizing symptoms in four quantitative studies [50, 62, 76, 77]. In a qualitative study, both autistic adolescents and caregivers indicated that suppression and withdrawal frequently led to outbursts, even after the initial emotional triggers had diminished [74].

#### The physiology of ER

Chiu et al. [51] found no significant differences in HRV between autistic and non-autistic adolescents. However, within the autistic group of average cognitive ability, poorer ER was more strongly associated with lower HRV. In an fMRI study, autistic adolescents with average cognitive ability showed heightened and sustained activation in the salience network, pulvinar, and dorsolateral prefrontal cortex in response to negative stimuli, which predicted ER difficulties [67].

#### Interventional studies

Six studies examined interventions based on cognitive behavioral therapy (CBT) and mindfulness techniques to enhance ER abilities in autistic adolescents [49, 52, 53, 56, 72, 75], and one investigated a non-adapted intervention [68]. Except for one study that included participants with mild cognitive difficulties [52], all interventions focused on adolescents with average cognitive abilities across a broad age range. A pilot study found that an individual psychotherapeutic intervention led to medium-to-large improvements in parent-reported ER from pre- to post-intervention, although self-reports did not reflect this change [56]. In a related study of the same intervention, improvements in dysphoric ER difficulties were predicted by the therapeutic alliance at different time points [49]. Three additional pilot studies assessed parallel group interventions for adolescents and parents, showing improvements in parent-reported ER, though effect sizes varied by measure and time point [53, 72, 75]. A school-based, group-format intervention led by special needs teachers improved teacher-reported ER compared to a waitlist control group, but lacked follow-up data and did not show corresponding effects in student self-reports [52]. In a pre–post service evaluation of a comprehensive DBT intervention, autistic adolescents reported reduced ER difficulties and self-harming behavior, with change scores similar to those of a non-autistic control group [68].

Five studies assessed group interventions where ER was a secondary outcome, including three RCTs [58, 59, 73] and two within-subject design pilot trials [63, 71]. Two of these evaluated interventions with parallel parent groups targeting social competencies in autistic adolescents; both studies found enhanced parent-reported ER post-intervention [58, 71], although only Fatta et al. [58] reported sustained effects at follow-up. Santomauro et al. [73] found that a CBT-informed depression intervention enhanced cognitive reappraisal, with self-reported improvements maintained at follow-up. Gerber et al. [59] reported that a digital, single-session CBT-based intervention targeting internalizing symptoms improved ER at follow-up, with greater effects in parent-reports than in self-reports. Kim et al. [63] reported conflicting feedback following an intervention for executive functions and daily living skills: adolescents reported improved ER, whereas parents noted worsening ER.

#### Comparison of autistic and non-autistic populations

Autistic adolescents, spanning a wide age range and predominantly with average cognitive abilities, consistently demonstrated more ER difficulties than both normative data [61, 70, 78–80] and non-autistic peers [51, 57, 65]. Two studies found no differences in the use of adaptive or non-adaptive ER strategies between autistic and non-autistic groups [64, 69], whereas one study, despite a smaller but comparable sample, reported greater use of maladaptive strategies among autistic adolescents [66].

#### Comparison of autistic and non-autistic clinical populations

One study found that autistic adolescents with average cognitive abilities had greater ER difficulties than peers with ADHD [57]. Another study reported that autistic adolescents had greater executive function problems related to ER than those with anorexia nervosa [77]; notably the autistic group in this study (*N =* 29) was all female and had a wide IQ range (42–145).

## Discussion

Given the significant burden of co-occurring psychiatric disorders among autistic adolescents [99] and the proposal that ER may serve as a transdiagnostic factor in the etiology of psychopathology [5], this systematic review specifically examined ER in autistic adolescents aged 10–24 years. While all included studies met this age criterion, most covered broad age ranges within this span and rarely provided age-stratified analyses, limiting our ability to examine ER differences across adolescent subgroups. This is, to our knowledge, the first review to synthesize both qualitative and quantitative data on ER in this population. The results led to the identification of four key themes reflecting our current understanding of ER in autistic adolescents, and two additional themes addressing the differences between autistic adolescents and their non-autistic peers.

### Factors associated with ER

Our analysis identified several key factors linked to ER in autistic adolescents. Better theory of mind and social skills were associated with more effective ER [57, 64, 73], while alexithymia and limited emotional awareness contributed to greater ER difficulties [61, 65, 74]. These findings highlight the importance of co-regulatory support during adolescence and are consistent with the extended process model of ER [12], suggesting that higher theory of mind and social skills facilitate the implementation stage, whereas alexithymia and limited emotional awareness hinder identification and monitoring.

The association between core ASD symptom severity and ER was mixed. Three studies found a positive association [54, 61, 80] supporting previous research [22, 33] and the biosocial model of emotion dysregulation [20]. In contrast, other studies found no association [66, 76], which may reflect differences in measurement, informants, or sample composition. Notably, positive associations were observed in average ability samples assessing ER on all process model stages, while null findings occurred in a sample with below average cognitive ability and moderate ER difficulties [76], and in a study focused on self-reported ER strategies.

Most studies found no effects of age and gender on ER abilities, differing from results in non-autistic populations [34]. However, these findings should be interpreted cautiously due to small samples and uneven gender distributions. One larger study noted a small age effect on gender differences, where older females exhibited higher dysphoria [78], which aligns with previous findings regarding age and female gender in non-autistic adolescents [100]. The predominance of cross-sectional studies with broad age ranges may have limited the ability to detect developmental trajectories in ER. Differences in informants and sample characteristics likely contribute to inconsistencies with non-autistic populations. Future research should prioritize longitudinal designs to clarify ER development and its relationship with gender over time.

### Associations between ER and psychopathology

In line with previous research [40], the included studies consistently showed that ER difficulties across all stages of the process model were associated with both internalizing and externalizing symptoms. This pattern was robust across diverse samples, spanning a range of cognitive abilities and ages, which supports the transdiagnostic role of ER in autistic adolescents. While the variability in cognitive ability and age enhances the generalizability of these findings, potential subgroup differences still require further investigation. Overall, these results highlight the complexity of ER as a transdiagnostic factor in psychopathology [5] and underscore the importance of targeting all stages of ER to improve mental health outcomes in this population [39].

### The physiology of ER

Two studies examined physiological aspects of ER in autistic adolescents. Sustained neural processing in the salience network—including the pulvinar and the dorsolateral prefrontal cortex—suggests difficulties disengaging from negative information, which may contribute to ER challenges in autism [67]. Lower resting HRV was also significantly associated with ER difficulties [51], indicating its potential as a biomarker for ER impairment and informing treatment approaches. Future research should include larger, more diverse samples and account for co-occurring psychiatric conditions.

### Interventions targeting ER in autistic adolescents

The reviewed studies provide valuable insights into interventions aimed at improving ER in autistic adolescents. Both CBT-based s and mindfulness interventions showed effectiveness, though effect sizes and sustainability varied [49, 52, 53, 56, 58, 59, 72, 73, 75]. Group-based programs with parallel parent sessions proved beneficial [53, 72, 75], highlighting the importance of engaging supportive environments during adolescence [101, 102]. Interventions involving parents or teachers may be particularly effective due to context-dependence of ER [11], and autistic individuals' difficulties in generalizing skills across settings [103]. The effectiveness of a non-adapted DBT program in a routine service setting [68] suggests broader applicability, supporting further investigation into autism-specific adaptations [104]. Moreover, interventions focusing on social skills [58, 71] and internalizing symptoms [59, 73] also improved ER, underscoring the interconnectedness of these constructs.

Despite these positive findings, there was considerable variability in effect sizes and follow-up outcomes, pointing to the need for consistent, long-term evaluation. Most studies had small samples of average-ability participants, and only three were RCTs [58, 59, 73], limiting generalizability. Discrepancies between informant reports [52, 56, 63] underscore the necessity for multi-method evaluation [35]. Including clinical outcomes, such as hospitalization rates [68, 75] may better establish the clinical relevance of interventions. Incorporating feasible physiological measures, such as salivary biomarkers, could further enhance intervention assessment [105, 106].

### Comparison of autistic and non-autistic populations

The reviewed studies consistently show that autistic adolescents experience greater challenges with ER compared to non-autistic and other clinical groups [51, 57, 61, 65, 70, 77–80]. Findings on adaptive and maladaptive strategy use were mixed: some studies found no differences [64, 69], while others reported greater use of maladaptive strategies among autistic adolescents [66]. These results highlight the complexity of ER and suggest that differences may exist in both the frequency and effectiveness of strategy use [12]. Future research should combine assessments of ER strategies with contextual information and direct measures of ER effectiveness to yield a more comprehensive understanding.

### Strengths and limitations

A key strength of this review is its rigorous methodology, including pre-registration, adherence to PRISMA guidelines [41], and a mixed-methods approach. Our explicit inclusion criteria—requiring validated autism diagnoses and a defined age range— ensured focused insights into ER in autistic adolescents. However, several limitations should be noted. First, autistic adolescents with cognitive impairment were significantly underrepresented, underscoring the need for research in this population [107]. Despite our mixed-methods approach, only two qualitative studies met our inclusion criteria, limiting perspectives from autistic lived experiences. Most studies were conducted in Western, Educated, Industrialized, Rich and Democratic (WEIRD) [108] societies, with limited reporting of racial or socio-economic diversity, which restricts generalizability. Finally, while our focus on the key developmental stage of adolescence is a strength, its expanded age range [27] is still broad and may mask developmental differences. The lack of age-stratified analyses in primary studies limited our ability to identify developmental patterns within adolescence. This reflects a wider gap in the field and underscores the need for more fine-grained, age-specific, and longitudinal research.

## Conclusion

This review highlights the central role of ER in both internalizing and externalizing symptoms among autistic adolescents. Consistent associations with core autism features and psychopathology reinforce ER’s importance as a transdiagnostic factor, with autistic adolescents exhibiting more ER difficulties than their non-autistic peers. Nonetheless, considerable uncertainties remain— particularly concerning developmental trajectories, gender differences, and neurobiological underpinnings.

The extended process model of ER [12] provides a valuable framework for organizing research and clarifying underlying mechanisms. Future studies would benefit from explicitly specifying the ER stage under investigation— identification, selection, implementation, or monitoring— and align measurement instruments accordingly. Qualitative research capturing autistic adolescents’ lived experiences is also needed to illuminate specific ER stages and inform tailored supports.

A major challenge is the heterogeneity of measurement approaches and discrepancies between self- and parent-reports. Studies variously assess ER strategies, difficulties, or aspects of executive function, complicating cross-study comparisons. Notably, parent-reports often indicate more severe ER challenges than self-reports, highlighting differences in perspective, insight, or context. Addressing these discrepancies will require multi-informant, multi-method approaches, and explicit investigation of their sources and implications. Integrating both adolescents and caregiver perspectives will provide a more nuanced and comprehensive understanding. There is also a clear need to incorporate physiological measures—such as heart rate variability or neural markers— to capture ER processes more objectively. Research addressing neural circuitry and physiological mechanism, as conceptualized in the RDoC framework, remains limited and warrants further attention.

Existing interventions show promise, but further research is needed to establish their long-term efficacy and broader applicability. Future priorities include longitudinal designs, greater sample diversity, and the integration of physiological biomarkers and real-world ecologically valid assessments to guide and personalize interventions [105]. These findings highlight the importance of routinely assessing and targeting ER in autistic adolescents—ideally using multi-informant methods and involving families or teachers—to support better mental health. Clinicians should prioritize individualized, context-sensitive interventions that address social skills, theory of mind, and emotional awareness, as early support for ER may help reduce psychiatric comorbidities in this population.

## Supplementary Information

Additional file 1. Quality Appraisal (MMAT) of included studies.

Additional file 2. PRISMA Checklist.

Additional file 3. Visualization of participants age ranges across main result themes.

## Data Availability

All data relevant to the study are included in the article or are available as supplementary information.

## Abbreviations

ASD: Autism Spectrum Disorder
CBT: Cognitive behavioral therapy
DBT: Dialectic behavioral therapy
ER: Emotion regulation
ED: Emotion dysregulation
EPM: Extended Process Model
HRV: Resting heart rate variability
MMAT: Mixed Methods Appraisal Tool
PRISMA: Preferred Reporting Items for Systematic Reviews and Meta-Analyses
RCT: Randomized controlled trial
RDoC: Research Domain Criteria
